# 

**Published:** 1895-03

**Authors:** 


### CONTENTS FOR MARCH, 1895.

ORIGINAL COMMUNICATIONS. PAGE

The Application of Intermittent Filtration to Domestic Filters. By GEORGE W. RAFTER ..... 449
Observations Upon an Anencephalic Monster. By E. B. ANGELL, M. D., and S. L. ELSNER, M. D ..... 462
Occipito-Posterior Positions. By P. W. VAN PEYMA, M. D ..... 466
A Neurological Bust. By WILLIAM C. KRAUSS, M. D ..... 470

CLINICAL REPORT.

A Case of Malignant Diphtheria Saved by Antitoxin, Indicating the Great Value of the New Remedy. By SAMUEL G. DORR, M. D ..... 473

REPORTS OF SOCIETIES.

The Eighth Periodical International Ophthalmological Congress ..... 476
Report of State Board of Medical Examiners ..... 488
Committee on Litter ..... 491

EDITORIAL.

State Examination for Medical License ..... 492
Topics of the Month ..... 495

Personal ..... 499
Obituary ..... 501
Society Meetings ..... 501
Medical College Notes ..... 502
Book Reviews ..... 503
Literary Notes ..... 511

